# Catalytic and Non-Catalytic Roles for the Mono-ADP-Ribosyltransferase Arr in the Mycobacterial DNA Damage Response

**DOI:** 10.1371/journal.pone.0021807

**Published:** 2011-07-18

**Authors:** Christina L. Stallings, Linda Chu, Lucy X. Li, Michael S. Glickman

**Affiliations:** 1 Department of Molecular Microbiology Washington University School of Medicine, St. Louis, Missouri, United States of America; 2 Gerstner Sloan Kettering Graduate School of Biomedical Sciences, Summer Undergraduate Research Program, New York, New York, United States of America; 3 Division of Infectious Diseases, Memorial Sloan Kettering Cancer Center, New York, New York, United States of America; 4 Immunology program, Sloan Kettering Institute, New York, New York, United States of America; University of Medicine and Dentistry of New Jersey, United States of America

## Abstract

Recent evidence indicates that the mycobacterial response to DNA double strand breaks (DSBs) differs substantially from previously characterized bacteria. These differences include the use of three DSB repair pathways (HR, NHEJ, SSA), and the CarD pathway, which integrates DNA damage with transcription. Here we identify a role for the mono-ADP-ribosyltransferase Arr in the mycobacterial DNA damage response. Arr is transcriptionally induced following DNA damage and cellular stress. Although Arr is not required for induction of a core set of DNA repair genes, Arr is necessary for suppression of a set of ribosomal protein genes and rRNA during DNA damage, placing Arr in a similar pathway as CarD. Surprisingly, the catalytic activity of Arr is not required for this function, as catalytically inactive Arr was still able to suppress ribosomal protein and rRNA expression during DNA damage. In contrast, Arr substrate binding and catalytic activities were required for regulation of a small subset of other DNA damage responsive genes, indicating that Arr has both catalytic and noncatalytic roles in the DNA damage response. Our findings establish an endogenous cellular function for a mono-ADP-ribosyltransferase apart from its role in mediating Rifampin resistance.

## Introduction

Mycobacteria are ubiquitous environmental and pathogenic bacteria that must withstand a range of stresses present in their respective habitats. In the case of pathogenic mycobacteria like *Mycobacterium tuberculosis* and *Mycobacterium leprae*, most of these stresses are derived from the host immune system, whereas for environmental mycobacteria like *Mycobacterium smegmatis*, these stresses may arise from toxins and chemicals secreted by other organisms, UV radiation, osmotic stress, and heavy metal pollutants. Many of these stresses cause damage to a range of macromolecules, including the bacterium's genomic DNA. DNA double strand breaks (DSBs) are the most dangerous form of DNA damage and represent the greatest threat to genome integrity and cell survival. Prior studies have provided evidence that DNA damage responses are important for intracellular pathogens, including mycobacteria [1], [2], [3], [4]. Thus, understanding DSB responses is critical for understanding how mycobacteria survive in diverse conditions and environments.

This manuscript describes the transcriptional response of *M. smegmatis* to DSBs and implicates the mono-ADP-ribosyltransferase Arr in this response. ADP-ribosylation is a reversible covalent modificationin which the ADP-ribose moiety of NAD^+^ is attached to its target [5]. Two evolutionarily related families of enzymes catalyze this reaction: mono-ADP-ribosyltransferases (ARTs) and poly-ADP-ribosyl polymerases (PARPs). ARTs are common to both prokaryotes and eukaryotes and transfer a single ADP-ribose to their targets [5]. In contrast, PARPs have been identified only in eukaryotes and archaebacteria and are able to sequentially transfer ADP-ribosyl groups to form polymersthat regulate many cellular processes including DNA repair [6], [7]. Although their primary sequences diverge, both eukaryotic and prokaryotic ADP-ribosyltransferases share similar catalytic mechanisms and a characteristic three-dimensional fold encompassing a common NAD^+^ binding core of 5 β-strands arranged as two adjoining sheets [8], [9], [10]. The only known target of *M. smegmatis* Arr-catalyzed ADP-ribosylation is rifampin, an antimicrobial agent that inhibits RNA polymerase (RNAP) [11], [12], [13]. Rifampin binds in a pocket of the RNAP β subunit deep within the DNA/RNA channel and inhibits transcription by directly blocking the path of the elongating RNA [14]. ADP-ribosylation of rifampinby Arrresults in inactivation of the drug,presumably by preventing its interaction with the RNAP [8], [14]. Arr is responsible for the relative resistance of *M. smegmatis* to rifampin in comparison to mycobacteria that do not express Arr. Other mycobacteria that encode Arr homologs include the pathogens *M. marinum* and *M. ulcerans* [8]. In contrast, *M. tuberculosis* does not encode an Arr homolog and is, therefore, more sensitive to rifampin, which is a first line agent in treatment of Tuberculosis.

Beyond its role in ADP-ribosylating rifampin, endogenous protein or small molecule targets of Arr have not been identified. The most well-characterized prokaryotic ADP-ribosyltransferases are secreted toxins, including diphtheria toxin, *Pseudomonas aeruginosa* exotoxins A and S, cholera toxin, pertussis toxin, and *Escherichia coli* LT-I and LT-II, which all target proteins in the host cellto facilitate pathogenesis [15]. However, *M. smegmatis* Arr lacks an obvious secretion signal and thus is expected tomodify targets within the mycobacterial cell. Endogenous mono-ADP-ribosylation, in which the ADP-ribosyltransferase and the protein to be modified originate from the same cell, has been described in *M. smegmatis* [16], but the identities of the modified proteins have not been determined and the physiologic role of ADP-ribosylation is unknown. The experiments presented herein describe the *M. smegmatis* DSB response and demonstrate a role for Arr in this response. These experiments provide a physiologic role for mono-ADP-ribosylation in mycobacteria apart from its function in rifampin resistance.

## Results

### Double strand DNA breaks induce a diverse and coordinated response in mycobacteria

To better understand the mycobacterial pathways that respond to double strand DNA damage, we used whole genome transcriptional profiling to detect changes in *M. smegmatis* gene expression during DNA double strand breaks (DSBs) caused by the I-SceI homing endonuclease [17], [18]. The analyses compared two *M. smegmatis* strains: mgm181 and mgm182. Anhydrotetracycline (ATc) treatment of both strains induces expression of a hemagglutinin (HA) epitope-tagged I-SceI homing endonuclease, which cuts at a single site engineered into the mgm182 genome. Thus, only the mgm182 genome is cleaved by I-SceI, while mgm181 expresses the endonuclease without suffering a chromosomal break. For the remainder of the paper we will refer to mgm182 asthe site(+) strain and mgm181 as the site(−) strain. The advantage of the I-SceI system is that the DNA damage induced is limited to DSBs, unlike similar microarray experiments that have been performed using genotoxins like UV radiation, which causes many different types of DNA damage as well as damage to other macromolecules [1], [19]. The comparison of the site(+) and site(−) strains also allows us to distinguish transcriptional effects of DSBs from secondary effects of ATc induction and enzyme expression. There is evidence that DNA damage responses and repair pathways differ between logarithmically growing and stationary phasemycobacteria, which is most likely due to replication and chromosome copy number [20]. Thus, to comprehensively characterize the responses to DSBs, we analyzed the transcriptional profile of cells expressing I-SceI in log as well as stationary growth phases.

**Figure 1:**
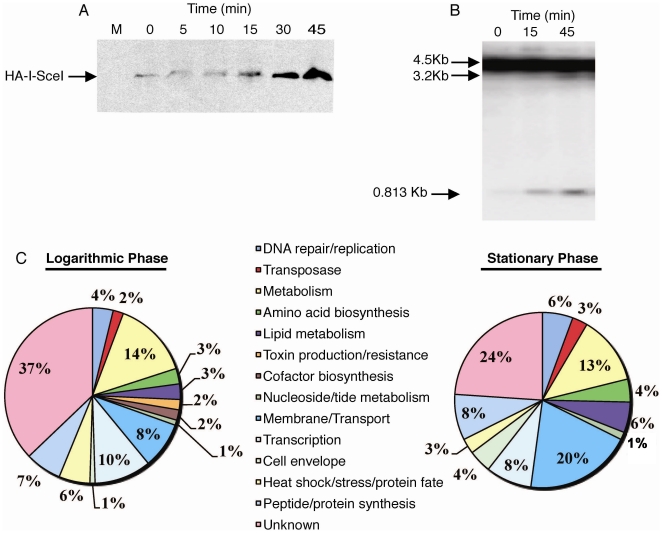
The *M. smegmatis* DSB response involves diverse classes of genes. A. Western blot analysis with HA specific antibodies of whole cell lysates of exponentially growing site(+) (mgm182) cultures to detect expression of HA-I-SceI before (T = 0) and at the indicated times after addition of 50 ng/ml ATc. M denotes the lane containing the molecular weight marker. Similar results were seen in all growth phases of site(−) and site(+) cultures. B. Southern blot analysis of SmaI digested genomic DNA from exponentially growing site(+) culturesbefore (T = 0) and at the indicated times after addition of 50 ng/ml ATc. Digested DNA was visualized with a radioactive probe that spans the I-SceI site. SmaI digestion without I-SceI site cleavage yields a 4.5 kb band. The I-SceI/SmaI double digest results in an 813 bp and 3.2 kb band. Similar results were seen in stationary phase site(+) cultures. C. Pie charts of the functional class distribution of genes that were upregulated 1.5 fold in site(+) but not in site(−) cultures following ATc treatment for 45 minutes, as compared to T = 0. Microarray experiments were performed with logarithmic and stationary phase cells, each with threebiological replicates and only genes with a p-value≤0.05 are included in this data set.

To detect HA-I-SceI expression, cultures were harvested before (T = 0) and after addition of ATc for protein extraction and western blot analysis with HA specific antibodies (Fig. 1A). To verify that ATc-induced HA-I-SceI expression causes chromosomal breaks in the site(+) strain, we prepared genomic DNA from cultures at the same time points as the protein analysis. I-SceI site cleavage was detected in the site(+) cultures by southern blotting using SmaI digested genomic DNA and a probe that spans the I-SceI site (Fig. 1B). As expected, DNA cleavage was not detected in site(−) (data not shown). The western and southern blot analyses showed that HA-I-SceI expression and chromosomal cleavage were induced to the maximal level we observed by 45 minutes of ATc treatment. These experiments also revealed that the ATc-inducible system used in these experiments was leaky enough that there is a basal amount of I-SceI enzyme expressionand recognition site cleavage in the site(+) strain even in the absence of ATc addition (Time = 0, Figs. 1A and B). Therefore, even at time zero, the site(+) cells are withstanding a low level of double strand DNA damage from basal I-SceI expression. Following the addition of ATc, the frequency of DSBs at the recognition site increases with the increase in I-SceI expression. At this point the only way for a cell to survive is to mutate the recognition sequence or lose I-SceI expression and/or activity [18].

To determine the transcriptional response to DSBs, we prepared cDNA libraries from either exponential or stationary phase site(−) and site(+) cultures before and after the addition of ATc. Fluorescently labeled cDNAs were co-hybridized to microarrays spotted 4 times with oligos representing 6746 *M. smegmatis* ORFs. Table S1 provides the ratio of transcript level in site(+)/site(−) for every gene represented on the microarray for each experiment. We first analyzed the genes that specifically responded to the increase in I-SceI generated DSBs after ATc addition by comparing the genes that were upregulated >1.5 fold in the site(+) strain and not in the site(−) strain following ATc treatment. Specifically, we found that 108 and 82 genes were upregulated in ATc treated log and stationary phase site(+) cells, respectively (Fig. 1C and Tables S2 and S3). In both growth phases, the upregulated genes represented a wide range of functional classes, with DNA repair pathway components making up less than 10% of the upregulated transcripts (Fig. 1C). Thus, the DSB response in *M. smegmatis* does not simply involve the regulation of DNA repair pathways, but is actually a coordinated response ofmany metabolic processes including transcription, translation, replication, sugar metabolism and lipid biogenesis. Most of the genes upregulated in either growth phase were genes of unknown function, highlighting the amount that remains to be characterized about the mycobacterial DNA damage response. Most functional classes of genes that were upregulated were evenly represented between stationary and log phase cells, except for membrane transport, lipid metabolism, and cell envelope pathways, which collectively made up 30% of the genes upregulated during DSBs in stationary cultures and only 12% in log phase cells.

Since we had observed a low level of I-SceI expression even in the absence of ATc (Figs. 1A and 1B), we also analyzed what genes were up or downregulated in site(+) cells at T = 0, as compared to the site(−) strain. These genes are responding to a low level of chronic chromosomal breakage that does not impair cell growth. Tables S4 and S5 list the genes that were upregulated during both low (−ATc, T = 0) and high (+ATc, T = 45) levels of DSBs in each growth phase. Table 1 summarizes the number of genes that responded to both levels of DSBs in log and stationary cultures. The most obvious result in Table 1 is the number of genes that respond to DSBs during stationary phase is much higher than in exponential growth phase. This implies that survival during the continuous attacks on the DNA requires more changes in the gene expression profile of stationary cells, whereas in log phase cells these factors are already expressed at ample levels. Proteins involved in DNA damage responses may be more abundant in exponential growing cells because of the high levels of DNA replication and transcription within these cells, which generate a certain amount of DSBs that must be resolved to maintain genomic integrity.

**Table 1:** Summary of I-SceI microarray data.

Growth Phase	Time of ATc treatment (min)	# of genes upregulated 2 fold in site(+)	# of genes downregulated 2 fold in site(+)
Log	0 and 45	33	2
Stationary	0 and 45	259	292
Log and Stationary	0 and 45	14	0

The microarray compares the transcriptional profile of two *M. smegmatis* strains, site(+) (mgm182) and site(−) (mgm181). The table summarizes the number of genes that were up or downregulated≥2× in log and/or stationary site(+) cultures as compared to site(−) cultures during both low (T = 0) and high (T = 45) levels of DSBs. Experiments were done in triplicate and all of the genes included in the table had a p value≤0.05.

### *M. smegmatis* ADP-ribosyltransferase Arr is upregulated in response to a range of stresses, including DNA damage

**Figure 2:**
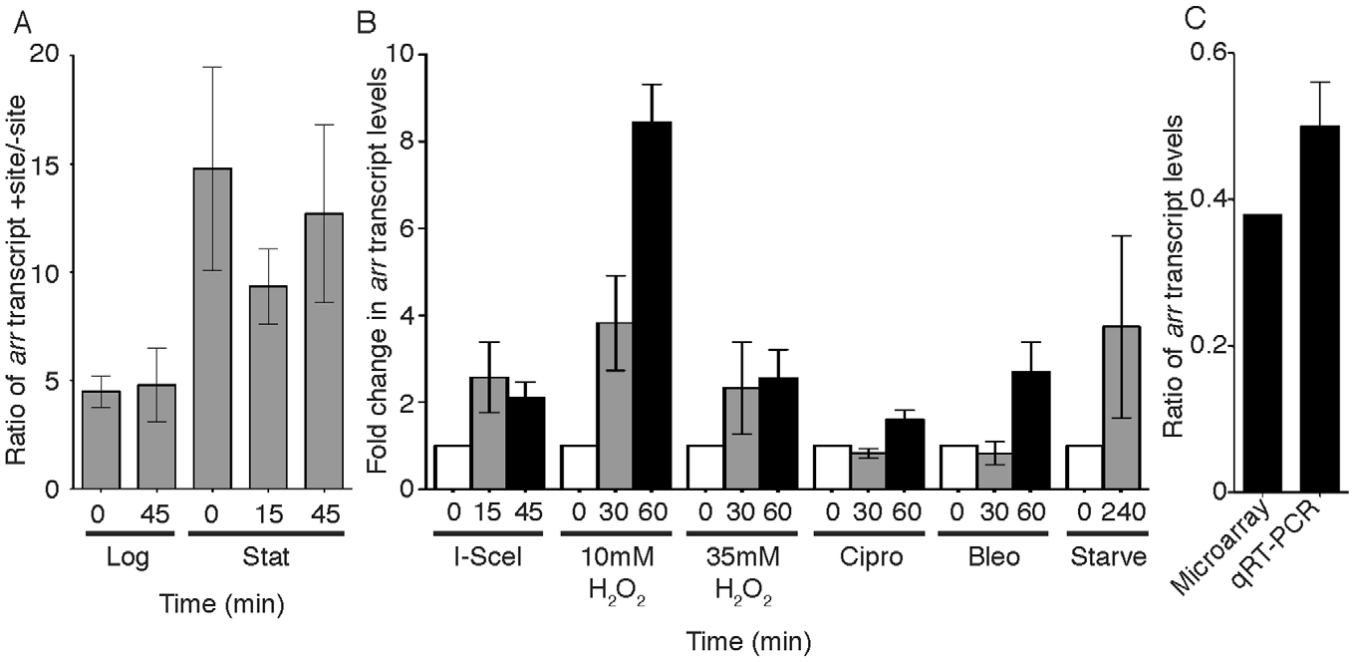
*arr* is transcriptionally upregulated in response to a range of stresses. All graphical data is represented as the mean ± SEM of biological triplicates. A. Mean ratios of *arr* transcript levels in site(+) versus site(−) cultures from microarray experiments. The time after ATc addition is indicated beneath the graphs for both log and stationary experiments. All p-values for *arr* transcript levels were <0.05 for the comparison of site(+) to site (−). B. Mean ratios of *arr* transcript levels in site(+) cultures following ATc addition (I-SceI bars) and wild-type *M. smegmatis* treated with hydrogen peroxide (H_2_O_2_), 10 µg/ml Ciprofloxacin (cipro), 10 µg/ml bleomycin (bleo), or starved in PBS +0.05% Tween 80 (starve), as compared to untreated cultures. Transcript levels were determined by qRT-PCR with primers specific for *arr* and normalized to *sigA* transcript levels. C. Mean ratios of *arr* transcript levels in *M. smegmatis* Tet-CarD cultures grown in the absence of ATc for 13 hours to deplete CarD versus levels in control cultures, as determined by either microarray (p-value<0.05) or qRT-PCR analysis.

The I-SceI microarray analyses revealed that 14 *M. smegmatis* genes were upregulated more than 2 fold in both log and stationary cultures during both low and high levels of I-SceI expression (Tables 1 and S6). The most highly upregulated gene in this group was *arr* (MSMEG_1221), which encodes a mono-ADP-ribosyltransferase and was transcriptionally upregulated as high as 15 fold in stationary cultures and 5 fold in logarithmic cells (Fig. 2A). The transcriptional induction of *arr* following double strand breaks was verified using quantitative real time PCR (qRT-PCR, Fig. 2B). The high level induction during DSBs, and the well established role of ADP-ribosylation in the eukaryotic DNA damage response, prompted us to investigate the role of Arr in the *M. smegmatis* DNA damage response. To verify that *arr* transcription is upregulated in the presence of DNA damage, we treated wild-type *M. smegmatis* cultures with bleomycin or ciprofloxacin and measured *arr* transcript levels before and after treatment by qRT-PCR. *arr* transcript levels increased by 60 minutes of treatment with either genotoxin(Fig. 2B). To determine if *arr* upregulation was specific for genotoxic stress or part of a general stress response, we treated wild-type *M. smegmatis* with hydrogen peroxide (oxidative stress) and nutrient deprivation before measuring changes in *arr* transcription by qRT-PCR (Fig. 2B). We found that *arr* transcription was as responsive to oxidative stress and starvation as it was to DNA damage, suggesting that Arris involved in a general stress response.

Besides *arr*, another gene that was transcriptionally upregulated more than 2 fold in log and stationary cultures during both low and high levels of I-SceI activity was previously characterized *carD* [17] (Table S6). We have shown that CarD is an essential mycobacterial protein that is required for survival during a range of stresses. Analysis of microarrays performed during CarD depletion [17] revealed that *arr* transcript levels are more than 2.5 fold lower in cells depleted of CarD (Fig. 2C). The downregulation of *arr* mRNA during CarD depletion was also verified using qRT-PCR (Fig. 2C). These data imply that *arr* is positively regulated by CarD, either directly or indirectly, which could explain their similar patterns of induction in response to genotoxic, oxidative, and nutrient deprivation stresses. These data suggest that CarD and Arr function in similar pathways.

### Arr requires substrate binding and catalytic activity to confer rifampin resistance in *M. smegmatis*

**Figure 3:**
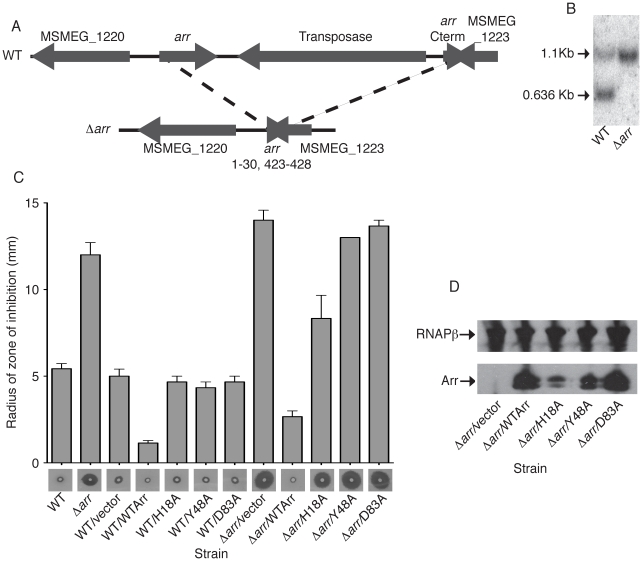
Arr requires substrate binding and catalytic activity to confer rifampin resistance in *M. smegmatis*. A. Schematic of the region of the *M. smegmatis* chromosome containing *arr* that was deleted via allelic exchange (nucleotides 1290611 to 1293058). The wild-type *arr* gene was replaced with a truncated null allele (Δ*arr*) containing only the first 10 and last 2 codons, thus yielding the *M. smegmatis* Δ*arr* strain. B. Southern blot analysis of wild-type *M. smegmatis* and Δ*arr* using PvuI digested genomic DNA and a probe that spans the 800 nucleotides upstream of the *arr* gene. PvuI digestion of wild-type *M. smegmatis* yields two bands of 1085 and 636 base pairs. PvuI digestion of Δ*arr* results in a 1085 bp and a 1067 bp band, which are indistinguishable on the southern blot. C. Wild-type *M. smegmatis* or Δ*arr* strains expressing wild-type FLAG-Arr, FLAG-tagged Arr point mutants, or containing an empty expression vector (vector) were plated as a lawn on LB. Disks soaked with 5 µl of a 25 mg/ml rifampin stock in DMSO were placed in the center of each lawn. Following two days of growth at 37°C, the radius of the zone of inhibition was measured. Graph shows the mean radius from three experiments. Photos of the zone of inhibition from a representative experiment are also shown for each strain. Disks soaked in DMSO with no drug produced no zone of inhibition (data not shown). D. Western blot of whole cell lysates with antibodies specific for the FLAG epitope tag or RNAPβ (loading control) from Δ*arr* cultures expressing FLAG-tagged Arr or FLAG-tagged Arr point mutants.

The only known target of Arr catalyzed ADP-ribosylation is rifampin, a chemical inhibitor of RNAP. To determine the role of Arr in stress responses, a function that is presumably independent of its function in rifampin resistance, we took a genetic approach and deleted *arr* from the *M. smegmatis* genome. *M. smegmatis arr* is flanked on the 3′ end by a predicted transposase and a duplication of the last 37 codons of *arr* (Fig. 3A) [8]. To delete both full-length *arr* and truncated *arr*, we replaced the region containing the *arr* gene, the transposase, and the *arr* duplication with a null *arr* allele containing only the first 10 and last 2 codons, thus yielding the *M. smegmatis* Δ*arr* strain (Fig. 3A). The truncated allele, which also resulted in a deletion of the last 3 codonsof the hypothetical gene MSMEG_1223, was confirmed by southern blotting (Fig. 3B). The Δ*arr* strain grew at the same rate as wild-type *M. smegmatis* and was not more sensitive to killing by the stresses used in the qRT-PCR experiments (data not shown).

Inactivation of *arr* leads to increased susceptibility of *M. smegmatis* to killing by rifampin [12]. We confirmed this phenotype in our Δ*arr* strain by measuring the zone of inhibition by a rifampin soaked disk on a lawn of either wild-type *M. smegmatis* or Δ*arr* (Fig. 3C). We were able to complement the rifampin sensitivity of Δ*arr* by expressing an N-terminally FLAG-tagged Arr protein (Figs. 3C and D). Expression of FLAG-Arr also increased the resistance of wild-type *M. smegmatis* to rifampin, further confirming that the tagged protein is functional (Fig. 3C).

Prior biochemical and structural studies identified three amino acid substitutions in Arr, H18A, Y48A, and D83A, that abolish its ability to ADP-ribosylate rifampin in vitro and to confer rifampin resistance in *E. coli* [8]. H18 and Y48 are predicted to contribute to NAD^+^ binding,whereas D83 is in the conserved substrate binding loop [8]. To investigate the role of these residues in Arr function in mycobacteria, we complemented the Δ*arr* strain with a plasmid directing the expression of wild type Arr, Arr(H18A), Arr(Y48A), or Arr(D83A), each with an N-terminal FLAG epitope. Overexpression of FLAG-tagged Arr proteins containing any one of these amino acid substitutions failed to confer rifampin resistance in Δ*arr* or wild-type *M. smegmatis*, even though all Arr alleles were expressed in Δ*arr* (Figs. 3C and D). These data demonstrate that the substrate binding and catalytic activity of Arr are necessary to mediate resistance of *M. smegmatis* to rifampin.

### Induction of DNA repair genesis independent of *arr*

**Figure 4:**
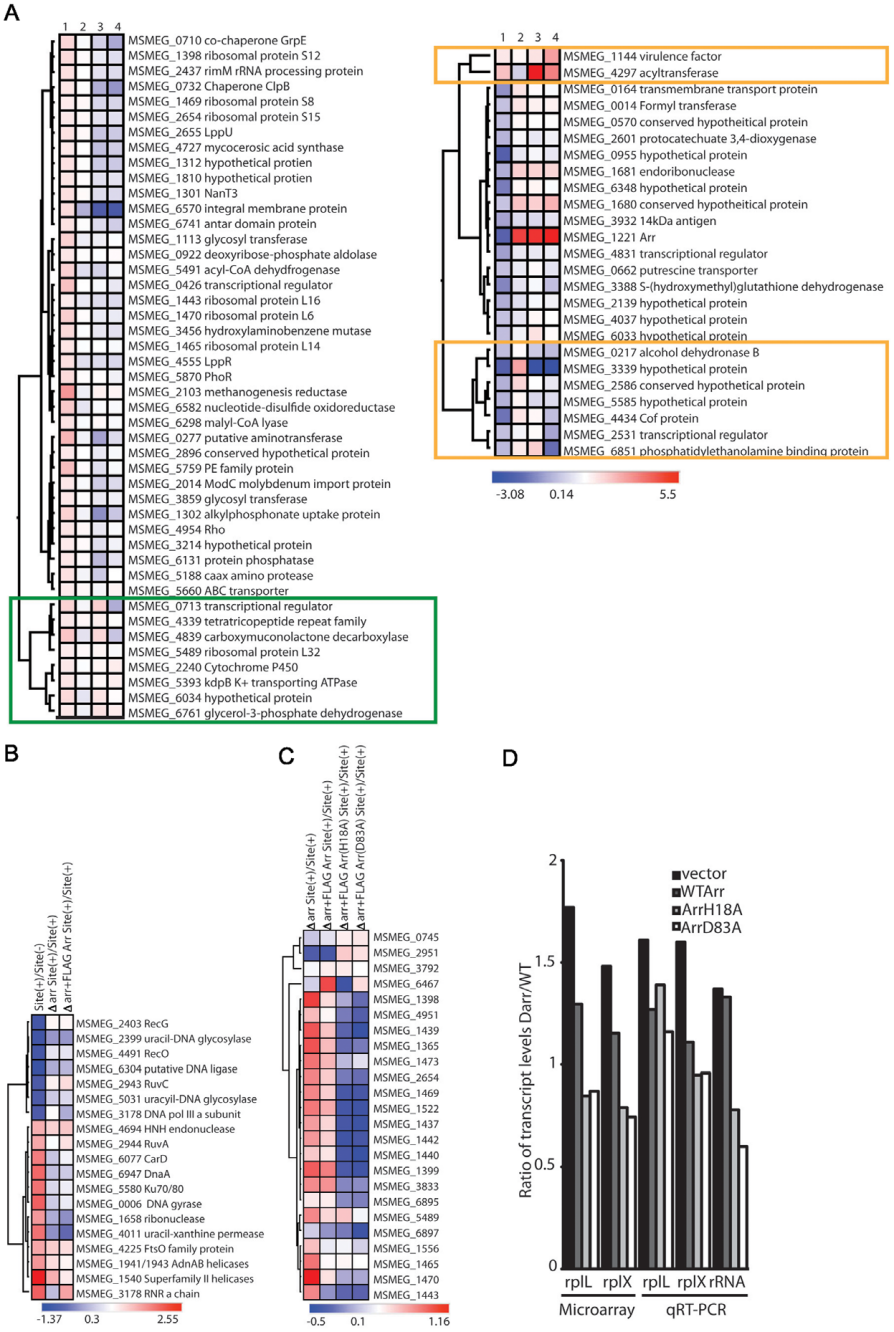
Catalytic and noncatalytic roles for Arr in the DNA damage response. Microarray and qRT-PCR experiments compared Δ*arr* site(+) strains expressing wild-type FLAG-Arr, FLAG-Arr(H18A), FLAG-Arr(D83A), or containing an empty expression vector (vector) to the original site(+) strain (mgm182) during exponential growth phase. All experiments were done in the absence of ATc when low levels of DSBs are occurring. A. The heat map shows the log_2_ ratio of transcripts that were up or downregulated 1.5× in Δ*arr* site(+) compared to wild type site(+) and complemented by expression of FLAG-Arr. Column 1 represents the ratio of Δ*arr*+vector site(+)/site(+), column 2 is Δ*arr*+FLAG-Arr site(+)/site (+), column 3 is Δ*arr*+FLAG-Arr(H18A) site(+)/site(+), and column 4 is Δ*arr*+FLAG-Arr(D83A)/site(+). The orange boxes highlight genes for which the up (2 genes) or downregulation (7 genes) observed in Δ*arr* cells (column 1) was not complemented by Arr(H18A) or Arr(D83A). The green box highlights genes in which the wild type expression pattern was restored by either Arr(H18A) or Arr(D83A). The heat maps only show genes that gave p values≤0.05. B. The heat map shows the log_2_ ratio of transcripts encoding DNA repair proteins that were up or downregulated 2× in site(+) compared to site(−). Column 1 is site(+)/site(−), column 2 is Δ*arr* site(+)/site(+), and column 3 is Δ*arr*+FLAG-Arr site(+)/site(+). The heat maps only show genes that gave p values≤0.05. C. The heat map shows the log_2_ ratio of ribosomal protein transcripts in Δ*arr* site(+) strains compared to wild-type site(+). Column 1 is Δ*arr*+vector site(+), column 2 is Δ*arr*+FLAG-Arr site(+), column 3 is Δ*arr*+FLAG-Arr(H18A) site(+), and column 4 is Δ*arr*+FLAG-Arr(D83A), each compared to wild-type site(+). The heat maps only show genes that gave p values≤0.05. D. Mean ratios of *rplL*, *rplX*, and 16S rRNA transcript levels in *M. smegmatis* Δ*arr* site(+) expressing wild-type FLAG-Arr, FLAG-tagged Arr point mutants, or containing an empty expression vector (vector) as compared to levels in original site(+) strain (mgm182) as determined by microarray or qRT-PCR. All experiments were done with exponential phase cultures in triplicate without ATc and therefore reflect basal expression of the I-SceI endonuclease equivalent to T = 0 in the microarray experiments.

To investigate the possible functions of Arr during the DNA damage response, we compared the gene expression profiles in the Δ*arr* strain to wild type during low levels of I-SceI generated DSBs. These microarray experiments compared three strains: *M. smegmatis* site(+), Δ*arr* site(+), and Δ*arr*+Flag-Arr site(+). We found numerous transcripts that were differentially expressed between Δ*arr* site(+) and wild type cells (Fig. 4A and Tables S7 and S10). We first examined the effects of deleting *arr* on genes encoding components of the DNA repair machinery that we had detected as up or down regulated during I-SceI induced DSBs in wild-type cells. We found that the regulation of these genes was preserved in the Δ*arr* strain (Fig. 4B). For example, the operon encoding the helicase-nuclease AdnAB (MSMEG_1941/1943 [21]) is induced in wild type cells following DSBs (induction ratio 2.31), as has been reported by others in *M. tuberculosis* following Mitomycin C treatment [22]. In Δ*arr* cells, this operon is still induced (Δ*arr*(site+)/wt(site+) = 1.5) indicating that *arr* does not participate upstream of these genes in the DNA damage response. Similar results were obtained for the other DNA repair genes induced by DSBs (Fig. 4B). These results indicate that Arr is a component of the transcriptional response to DNA damage, but not an upstream regulator of transcription of the DNA repair machinery.

### Arr suppresses ribosomal protein and rRNA levels in response to DNA damage

We next analyzed the full complement of genes up or downregulated in Δ*arr* cells compared to wild type after DSBs. After filtering on those genes with p values≤0.05 from the triplicates performed for each experiment, we found that 47 genes were >1.5 fold upregulated (Table S7) and 23 genes were <1.5 fold downregulated (Table S10) in Δ*arr* site(+) compared to wild type site(+) and complemented by expression of FLAG-Arr (Fig. 4A). Among the transcripts that were upregulated in the Δ*arr* during DNA damage were those encoding 7 ribosomal protein genes (Table S7). This pattern of overexpression of ribosomal components was also seen in our prior analysis of gene expression during CarD depletion [17]. Additional qRT-PCR experiments confirmed that *rplX* and *rplL*, encoding ribosomal protein L24 and L12 respectively, were overexpressed in the Δ*arr* strain compared to wild type during I-SceI induced DSBs (Fig. 4D). We also found that the 16S rRNA was upregulated in Δ*arr* during DSBs (Fig. 4D). The ribosomal protein transcript upregulation was complemented by a wild-type copy of Arr (Fig. 4C and 4D). These results suggest that one role of *arr* during the DNA damage response may be to regulate the expression levels of genes encoding components of the translation machinery, a function similar to CarD.

### Role of substrate binding and catalytic activities of Arr during DNA damage

The only known substrate for the Arr ADP-ribosyltransferase is the antibiotic rifampin. The results presented above imply a role for *arr* in the DNA damage response, a role that may involve ADP-ribosylation of protein targets in the mycobacterial cell. At present, no endogenous ribosylation targets are known in mycobacteria. To investigate whether the substrate binding and catalytic activities of Arr were required for its effects on gene expression during DNA damage, we performed microarray experiments with Δ*arr* site(+) strains expressing either FLAG-Arr(H18A) or FLAG-Arr(D83A), which fail to confer rifampin resistance (Fig. 3). Of the 70 genes significantly changed in the Δ*arr* strain, only a small number required the catalytic and substrate binding functions to restore wild type expression levels (Tables S8,S9,S11,S12). Specifically, 9 genes that were misregulated in the Δ*arr* strain compared to wild type (7 underexpressed and 2 overexpressed) were restored in the complemented strain but remained deregulated in the Arr(H18A) and Arr(D83A) strains (Fig. 4A, orange boxes). 8 genes required either the catalytic or the substrate binding activity of Arr for regulation (Fig. 4A, green box). In contrast, most genes that were deregulated in the Δ*arr* strain during DNA damage were restored to their wild type expression level by both Arr and either of the mutant *arr* alleles (Fig. 4A). In particular, expression of Arr(H18A) or Arr(D83A) suppressed most of the elevated ribosomal protein transcripts in the Δ*arr* strain to the same degree as wild type Arr (Figs. 4C and D, Table S13). These results demonstrate that the expression of Arr during DNA damage has both positive and negative effects on gene expression, but only a small set of genes require Arr catalytic activity for regulation during the mycobacterial response to DSBs.

### Arr associates with the RNAP and the stringent response regulator ribosomal protein L11

**Figure 5:**
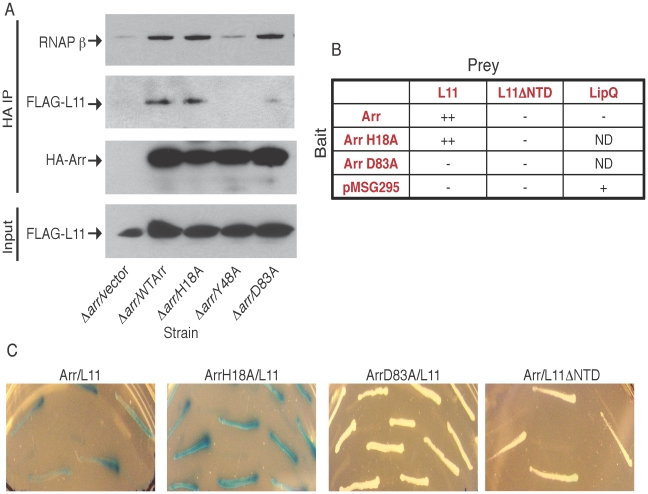
Arr interacts directly with the ribosomal protein L11. A. Western blot analysis of immunoprecipitation experiments with HA antibody from whole cell lysates of *M. smegmatis* Δ*rplK*::FLAG-L11 strains expressing HA-Arr or the indicated HA-Arr point mutants. FLAG specific antibodies were used to detect FLAG-L11 in inputs and eluates. HA specific antibodies and monoclonal antibodies specific for the RNAP β subunit were used to visualize HA-Arr and RNAP β in the eluates. B. Table summarizing the interaction data from yeast two-hybrid experiments. pMSG295 and LipQ were used as controls. ND = not done. A single + denotes that the interaction was detected, and two + symbols means the two proteins strongly interacted, based on visual inspection of the intensity of blue color on agar media containing X-gal. C. Photos from one experiment of diploid yeast cells containing bait and prey plasmid of the indicated combinations on plates containing Xgal, where a blue colony color represents a positive interaction.

To determine how Arr was affecting transcription, we chose to explore proteins that physically interact with the Arr protein. Since Arr has effects on the transcriptional profile during DNA damage, is able to ADP-ribosylate rifampin (a molecule that binds the RNAP [8], [14]), and some phages ADP-ribosylate RNAP, our first hypothesis was that Arr would be associated with the RNAP. To test this hypothesis, we immunoprecipitated whole cell lysates from Δ*arr* strains complemented with plasmids encoding HA-Arr, HA-Arr(H18A), HA-Arr(Y48A), or HA-Arr(D83A) with HA antibodies. Analysis of the eluates by western blotting with an antibody specific for the RNAP β subunit showed that Arr coprecipitates the RNAP β subunit (Fig. 5A). This association was preserved between RNAP β and Arr(D83A), the substrate binding mutant, and Arr(H18A), the catalytic mutant (Fig. 5A).

In previous immunoprecipitation experiments, we have shown that CarD associates with RNAP [17] through its interaction with the β subunit. In addition to the RNAP subunits, we found that CarD co-precipitated the ribosomal protein L11, but yeast two hybrid experiments failed to detect a direct interaction between CarD and L11 (data not shown) [17]. Prokaryotic ribosomal protein L11 is encoded by the *rplK* gene, which was originally designated as *relC* when a mutant of this gene in *E. coli* was identified as having a relaxed phenotype [23], [24]. Relaxed bacterial strains are unable to downregulate rRNA during the stringent response. The stringent response involves the production of (p)ppGpp, in part by the enzyme RelA, during nutrient, phosphate, and nucleotide deprivation, stationary phase, oxidative stress and alkaline shock. (p)ppGpp synthesis represses transcription of ribosomal components as well as directly inhibits DNA replication and other cellular processes [17], [25], [26], [27], [28], [29], [30], [31], [32]. In *E. coli*, L11 is necessary for RelA catalyzed synthesis of (p)ppGpp and in *E. coli*, *Bacillus subtilis*, *Thermus thermophilus*, and *Streptomyces sp*., stringent coupling between translation and transcription is eliminated by mutations in *rel* genes [23], [24], [33], [34], [35], [36], [37], [38], [39]. The importance of the RelA protein in the mycobacterial stringent response is well-established [25], [40], [41], but the role of mycobacteria L11 has yet to be investigated.

Because of their co-association with RNAP during co-immunoprecipitation experiments and their roles in regulating transcription of the translation machinery, we were curious about whether L11 and Arr interacted physically. To explore this question, we replaced the endogenous copy of *M. smegmatis rplK* with a gene coding for an N-terminally FLAG-tagged L11 protein in our strains expressing HA-Arr. The *M. smegmatis* Δ*rplK*::FLAG-L11 strains grew like wild-type, indicating that the FLAG-tagged version of L11 was functional because L11 is predicted to be an essential gene [42], a prediction we confirmed by our inability to obtain a L11 null strain by specialized transduction (data not shown). Co-immunoprecipitation experiments in Δ*rplK*::FLAG-L11 strains expressing HA-Arr or HA-Arr point mutants showed that wild-type Arr and Arr(H18A) were able to co-precipitate similar amounts of FLAG-L11, whereas Arr(D83A) and Arr(Y48A) were not (Fig. 5A). These results indicate that L11 associates with Arr, and that this association is dependent on the predicted substrate binding residues of Arr. To determine if this interaction was direct, we performed yeast two-hybrid experiments between L11 and Arr. The results of these assays, summarized in Figure 5B and shown in Figure 5C, demonstrated that L11 and Arr interacted specifically and directly with each other. Arr(H18A), but not Arr(D83A), was also able to interact with L11 in the yeast two-hybrid experiments, supporting the *M. smegmatis* co-immunoprecipitation results (Fig. 5). We also tried testing the Arr(Y48A) point mutant in the yeast two-hybrid experiments, but, unlike with the other constructs, we could not detect expression of Arr(Y48A) in yeast (data not shown). This mutant also did not co-precipitate the RNAP or L11 from *M. smegmatis* lysates, together suggesting that Arr(Y48A) may be structurally unstable or unfolded (Fig. 5A).

In *E. coli*, RelA catalyzed (p)ppGpp synthesis is dependent on the N-terminus of L11 and deletion or mutation of this region is enough to confer a relaxed phenotype [43], [44]. The N-terminus of L11 is actually sufficient to activate RelA in a ribosome-dependent manner even though this region alone is incapable of associating with the ribosome and thus must be functioning *in trans* [45]. To determine if the N terminal region of mycobacterial L11 directly interacts with Arr, we tested a truncation of L11 missing the N-terminal 33 amino acids (L11ΔNTD) in the yeast two-hybrid assays. L11ΔNTD did not interact with Arr (Figs. 5B and 5C), indicating that Arr interacts with the N-terminus of L11, which overlapswith the functional domain involved in the stringent response in *E. coli*.

Unfortunately,despite extensive efforts, we were unable to demonstrate ADP-ribosylation of L11 by Arr in vitro using NAD that was labeled with ^32^P, biotin, or etheno as a cofactor.However, these studies were complicated by the relative instability of purified L11 protein (data not shown). Thus, although Arr interacts directly with the N terminus of L11, we were unable to conclusively determine whether L11 is an ADP-ribosylation target of Arr.

## Discussion

### Regulating the transcription of the translation machinery in response to stress

A total of 14 genes were consistently upregulated more than 2 fold in *M. smegmatis* log and stationary cultures in response to both low and high levels of DNA damage. So far, we have described physiological functions for two of these genes, *arr* and *carD*, in regulating the levels of transcription of the translation machinery. Taken together, these studies indicate that a major response to double strand DNA damage is the induction of pathways that coordinately repress expression of the translational machinery. These findings highlight that stringent response-like changes in transcription are not only important in the classical case of nutrient deprivation, but also during double strand DNA damage.

### The role ofARTs in regulating transcription and translation

We have shown that Arr associates with the RNAP and the ribosomal protein L11 and affects the transcript levels of the translation machinery during DNA damage. Arr is not the first mono-ADP-ribosyltransferase proposed to modulate transcription and translation. One of the most well characterized bacterial exotoxins, Diphtheria toxin, ADP-ribosylates host elongation factor Tu, inactivating it and shutting down host protein synthesis. Evidence for endogenous ADP-ribosylation affecting transcription and translation comes from T4 and related bacteriophages. Infection of *E. coli* by these bacteriophages leads to the expression of three phage mono-ADP-ribosyltransferases, Alt, ModA, and ModB. Each phage ADP-ribosyltransferase modifies a distinct group of host proteins to regulate gene expression during the transition from host to phage protein synthesis. Alt is a structural component of the phage head that ADP-ribosylates one of the RNAPα subunits to enhance transcription from T4 early promoters [46], [47]. In a second step, ModA targets both α subunits of the host RNAP to reduce and stop transcription from T4 early promoters [48], [49], [50]. Alt has also been shown to ADP-ribosylate the RNAP β, β′, and σ subunits as well as trigger factor, EF-Tu, and the protein chaperone GroEL [51], [52]. ModB ADP-ribosylates ribosomal protein S1, EF-Tu, and trigger factor to directly affect host translation [49], [52].The genes encoding many Arr homologs, including *M. smegmatis arr*, are either on mobile genetic elements or clustered with predicted transposase encoding genes, which suggests acquisition from horizontal gene transfer [8]. One possibility is that *arr* genes were derived from lysogenic phages incorporated into their bacterial hosts. If this were the case, it would be expected that phage and prokaryotic ADP-ribosyltransferases would have similar specificities.

### ADP-ribosylation and the stringent response

The induction of ribosomal protein and ribosomal RNA in the Δ*arr* strain during DNA damage and the association of Arr with the RNAP and L11 suggest a role for Arr in the mycobacterial stringent response. There is precedencein other bacteria for ADP-ribosylation during starvation and sporulation, processes tightly linked to the stringent response. ADP-ribosyltransferase activity has been reported to increase in *Bacillus subtilis*, *Myxococcusxanthus*, and *Streptomyces species* in response to adverse environmental conditions that may involve severe DNA stress, nutrient limitation and sporulation [53], [54], [55], [56]. Studies with inhibitors of ADP-ribosylation have demonstrated that this activity is necessary for sporulation in *B. subtilis* [54] and when the stringent response is induced in *B. subtilis*, (p)ppGpp levels increase, GTP levels decrease, sporulation is initiated, and ADP-ribosyltransferase activity increases [57], [58], [59]. In addition, some ADP-ribosylation during sporulation is actually dependent on RelA and the stringent response [54].

However, our studies in *M. smegmatis* showed that Arr mutants deficient in binding L11 retain the ability to repress rRNA and ribosomal protein gene transcription, which suggests that Arr does not need to bind L11 to downregulate these transcripts. We were also unable to demonstrate ADP-ribosylation of L11 by Arr. Therefore, although Arr and L11 may both function to regulate transcription of the translation machinery, this function may not require them to interact physically. This implies that additional components of the Arr and L11 pathways are unaffected by the Arr(D83A) mutation. Arr substrate binding and catalytic activity mutants retain their ability to associate with the RNAP, and thus could conceivably be regulating transcription through this interaction, apart from L11.

### Implications for treatment of disease

Predicted ADP-ribosyltransferase genes have been identified in the genomes of numerous pathogenic mycobacteria including *M. ulcerans*, *M. marinum*, and *M. avium* [8], [60]. In addition, Arr homologs are present in other bacterial pathogens, including *Pseudomonas aeruginosa*, *E. coli*, *Klebsiella pneumoniae*, *Vibrio cholerae*, and *Acinetobacter baumannii* [8], [61], [62], [63], [64]. Further investigations are necessary to determine if the ADP-ribosyltransferases in these organisms function to withstand stresses imposed by the host. If so, targeting these enzymes could prove valuable as a treatment strategy by compromising the pathogen's ability to defend itself against host attacks and, in the case of enzymes with similar specificity as Arr, facilitating the use of rifampin because inhibition of Arr would sensitize these organisms to Rifampin. Endogenous ADP-ribosylation activity has also been reported in *M. tuberculosis*, but this activity is clearly not originatingfrom an enzyme with the exact same specificity as *M. smegmatis* Arr since wild-type *M. tuberculosis* remains sensitive to rifampin [16] and no clear homologue of Arr is present in the *M. tuberculosis* chromosome. Identification of novel ARTs in organisms like *M. tuberculosis* is hindered by the low sequence similarity between the ADP-ribosylating enzymes, but is worth pursuing as it could potentially provide exciting new approaches to treatment.

## Methods

### Media and Strains

All *M. smegmatis* strains were isogenic to mc^2^155 and were grown at 37°C in LB supplemented with 0.5% dextrose, 0.5% glycerol, and 0.05% Tween 80 (broth). MGM181, MGM182, and Tet-CarDstrains were described previously [17], [18]. The *arr* gene (MSMEG_1221) was deleted from the *M. smegmatis* genome via an allelic exchange system [65] that replaced the wild-type *arr* gene with a truncated null allele containing only the first 10 and last 2 codons, thus yielding the *M. smegmatis* Δ*arr* strain (*M. smegmatis* nucleotides 1290611 to 1290611 were deleted). The truncated allele conserves the reading frame of the wild type gene to avoid polar effects on neighboring genes that may be caused by the deletion.

Point mutations in *arr* were made by overlap extension PCR. The residue designations are based on the annotation of *arr* from J. Craig Venter Institute Comprehensive Microbial Resource. Our amino acid residue numbers are shifted by one residue from the published description of these mutants [8], but refer to the same amino acids. FLAG-tagged wild-type Arr and FLAG-tagged Arr point mutants were cloned into and expressed from a MOP promoter in pmsg383 (episomal, hygR). HA-tagged wild-type Arr and HA-tagged Arr point mutants were cloned into and expressed from a GroEL promoter in pmv261.Kan (episomal, kanR). Δ*rplK*::FLAG-L11 was engineered using a specialized transducing phage [66] to replace the endogenous *rplK* gene with a FLAG-tagged version followed by a hygR cassette.

### Antibiotics and chemicals

In mycobacteria, 20 µg/ml kanamycin, 50 µg/ml hygromycin, and 50 ng/ml of ATc were used. H2O2 (Fisher Scientific), MMS (Sigma), bleomycin(Alexis Corporation, San Diego, Ca), Ciprofloxacin (USB), and rifampin (Sigma) were used at the indicated concentrations.

### Antibodies

Primary antibodies include monoclonal antibodies specific for HA (HA11, Covance, Berkeley, Ca) FLAG (Clone M2, Sigma), and RNAP β (clone 8RB13, NeoClone, Madison, Wi).

### Microarrays

RNA was prepared from 20 ml of mycobacteria cultures with TRIzol (Invitrogen), bead beating 3× (FastPrep, Thermo Scientific), and QIAGEN's RNAeasy RNA clean-up protocol. The RNA was reverse transcribed into cDNA with the Stratagene Fairplay kit. cDNA coupled to fluorescent dyes Cy3 and Cy5 (GE Healthcare) were hybridized to gene chips spotted four times with oligos representing 6746 *M. smegmatis* ORFs (obtained from the J. Craig Venter Institute through the Pathogen Functional Genomics Resource Center). Experiments were done in triplicate and results were processed with GenePix software and analyzed in either GeneSpring GX or Partek Genomics Suite. Clustering was performed using the hierarchical clustering module on the Gene pattern server http://www.broadinstitute.org/cancer/software/genepattern/index.html.

### qRT-PCR

RNA was prepared from 5 ml of mycobacteria cultures with TRIzol and bead beating. To assure that there was no contaminating genomic DNA, RNA preparations were treated with Ambion's TURBO DNA-free kit. cDNA was created with Invitrogen's SuperScript III First-Strand Synthesis System. Quantitative PCR was performed with NEB's DyNAmo SYBR Green qPCR kit. All qRT-PCR experiments were done in triplicate.The following primer sequences were used for qRT-PCR. *M. smegmatis sigA* -5′-TGCCGATCTGCTTGAGGTAGG-3′ and 5′-TTCGTGTGGGACGAGGAAGAG-3′; *M. smegmatis arr*- 5′-GGCGCATCATGAACCACGTC-3′ and 5′-CTTCACCGGCAGCAAGTTCG-3′; *M. smegmatis rplL*- 5′-GCAGTCGGAATTCGACGTCATCCTC-3′ and 5′-CGAGATCCTTGGCTTCCTTCAGGC-3′; *M. smegmatis rplX*- 5′- GTGAACCGGATCAAGAAGCACACC-3′ and 5′- GAATCGACCACCATCACGTTCGAG-3′; *M. smegmatis* 16S rRNA- 5′-GTGCATGTCAAACCCAGGTAAGG-3′ and 5′-GGGATCCGTGCCGTAGCTAAC-3′.

### Immunoprecipitation and western blotting

For immunoprecipitation, 50 ml cultures were washed and lysed in 500 µl NP-40 buffer (10 mM sodium phosphate [pH 8.0], 150 mM NaCl, 0.25% NP-40, and Roche Complete protease inhibitor cocktail) by bead beating. 25 µl of lysate was used for input sample and the rest was added to monoclonal anti-HA agarose (Sigma) and rotated overnight at 4°C. The matrix was washed 3× with NP-40 buffer and proteins were eluted with 50 mM Tris-HCl [pH 7.5], 50 mM NaCl, 500 µg/ml HA peptide (Roche), and protease inhibitors. For western blot analysis, cells were lysed in 100 µl of NP-40 buffer by bead beating three times and adding lysates directly to SDS-PAGE loading buffer.

### Yeast Two Hybrid

Yeast Two Hybrid interaction studies were done as previously described [67]. Briefly, the bait plasmid comprised a fusion of the LexA DNA binding domain (BD) encoded in pEG202 to the N-terminus of Arr. The prey plasmid comprised a fusion of the LexA activation domain (AD) in pJSC401 to L11. The interaction between Arr and L11 was tested by mating of *Saccharomyces cerevisiae* strain EGY48 containing the AD fusion plasmid to strain W303a containing the BD bait plasmid and the *lacZ* reporter plasmid pSH18-34.pMSG295 and LipQ were used as controls for a positive interaction with each other, and a negative interaction with L11 or Arr [18].

## Supporting Information

For Tables S1–S6 the gene annotations are based on the older annotations from TIGR. New annotations for each gene may be found at the J. Craig Venter Institute Comprehensive Microbial Resource website. For tables S7–S13, the newer annotations are shown

Table S1 **I-SceI microarray- Ratio of** ***M. smegmatis*** **gene transcript levels in mgm182 (site(+)) as compared to mgm181 (site(−)) at both growth phases and all timepoints.**
(XLSX)

Table S2 **I-SceI microarray- Genes organized by functional category that are upregulated 1.5× only in the site(+) strain, and not in site(−), during log growth following ATc addition.** The increase in transcript levels is expressed as a ratio of levels at T = 45/T = 0. p-value≤0.5.
(XLSX)

Table S3 **I-SceI microarray- Genes organized by functional category that are upregulated 1.5× only in the site(+) strain, and not in site(−), during stationary (stat) growth following ATc addition.** The increase in transcript levels is expressed as a ratio of levels at T = 45/T = 0. p-value≤0.5.
(XLSX)

Table S4 **I-SceI microarray- Genes upregulated 2× throughout all timepoints in log phase site(+) cultures as compared to site(−). p-value≤0.5.**
(XLSX)

Table S5 **I-SceI microarray- Genes upregulated 2× throughout all timepoints in stationary site(+) cultures compared to stationary site(−) cultures. p-value≤0.5.**
(XLSX)

Table S6 **I-SceI microarray- Genes upregulated 2× in both log and stationary site(+) cultures at all timepoints, as compared to site(−).**
(XLSX)

Table S7 **Δ** ***arr*** **I-SceI microarray- Genes upregulated 1.5× in Δ** ***arr*** **site(+) as compared to wild-type site(+) and complemented by WT Arr expression, p value<0.05.**
(XLSX)

Table S8 **Δ** ***arr*** **I-SceI microarray- Genes upregulated 1.5× in Δ** ***arr*** **site(+) as compared to wild-type site(+) and complemented by WT Arr expression but not by expression of ArrH18A, p value<0.05.**
(XLSX)

Table S9 **Δ** ***arr*** **I-SceI microarray- Genes upregulated 1.5× in Δ** ***arr*** **site(+) as compared to wild-type site(+) and complemented by WT Arr expression but not by expression of ArrD83A, p value<0.05.**
(XLSX)

Table S10 **Δ** ***arr*** **I-SceI microarray- Genes downregulated 1.5× in Δ** ***arr*** **site(+) as compared to wild-type site(+) and complemented by WT Arr expression, p value<0.05.**
(XLSX)

Table S11 **Δ** ***arr*** **I-SceI microarray- Genes downregulated 1.5× in Δ** ***arr*** **site(+) as compared to wild-type site(+) and complemented by WT Arr expression but not by expression of ArrH18A, p value<0.05.**
(XLSX)

Table S12 **Δ** ***arr*** **I-SceI microarray- Genes downregulated 1.5× in Δ** ***arr*** **site(+) as compared to wild-type site(+) and complemented by WT Arr expression but not by expression of ArrD83A, p value<0.05.**
(XLSX)

Table S13 **Δ** ***arr*** **I-SceI microarray- Ribosomal protein transcript levels in Δ** ***arr*** **I-SceI microarray experiments.**
(XLSX)
